# Grasping without Sight: Insights from the Congenitally Blind

**DOI:** 10.1371/journal.pone.0110175

**Published:** 2014-10-10

**Authors:** Kayla D. Stone, Claudia L. R. Gonzalez

**Affiliations:** The Brain in Action Laboratory, Department of Kinesiology, University of Lethbridge, Lethbridge, Alberta, Canada; University of Turin and the Italian Institute of Technology, Italy

## Abstract

We reach for and grasp different sized objects numerous times per day. Most of these movements are visually-guided, but some are guided by the sense of touch (i.e. haptically-guided), such as reaching for your keys in a bag, or for an object in a dark room. A marked right-hand preference has been reported during visually-guided grasping, particularly for small objects. However, little is known about hand preference for haptically-guided grasping. Recently, a study has shown a reduction in right-hand use in blindfolded individuals, and an absence of hand preference if grasping was preceded by a short haptic experience. These results suggest that vision plays a major role in hand preference for grasping. If this were the case, then one might expect congenitally blind (CB) individuals, who have never had a visual experience, to exhibit no hand preference. Two novel findings emerge from the current study: first, the results showed that contrary to our expectation, CB individuals used their right hand during haptically-guided grasping to the same extent as visually-unimpaired (VU) individuals did during visually-guided grasping. And second, object size affected hand use in an opposite manner for haptically- versus visually-guided grasping. Big objects were more often picked up with the right hand during haptically-guided, but less often during visually-guided grasping. This result highlights the different demands that object features pose on the two sensory systems. Overall the results demonstrate that hand preference for grasping is independent of visual experience, and they suggest a left-hemisphere specialization for the control of grasping that goes beyond sensory modality.

## Introduction

We execute hundreds of reaching and grasping movements a day. Both vision and hapsis (i.e. sense of touch) play an integral role in guiding these movements. For example, when reaching for an object, such as an apple, both visual and haptic cues are needed to successfully complete the movement. Initially vision is used to identify the apple (e.g. shape, size, colour) and hapsis is used to assess properties such as object weight, temperature, and texture [1]–[3]. Numerous studies have reported a preference to use the right hand during visually-guided grasping [4]–[11], so it is likely one would choose this hand to pick up the apple. However, suppose you are searching for your keys in a bag, or a glass of water in the middle of the night. In these scenarios, only haptic cues would be used and essential to locate, recognize, and grasp the objects. Would the right-hand preference seen during visually-guided grasping persist in the absence of vision? To answer this question, Stone and Gonzalez [12] asked sighted and blindfolded participants to reach for and grasp blocks scattered on a tabletop in order to construct 3D models. The results showed that participants used their right hand significantly more to grasp the blocks while sighted, but even so, a preference to grasp with the right hand persisted during the blindfolded trials. However, if these blindfolded trials were preceded by five minutes of haptic experience (without vision), equal use of the right and left hands was observed [12]. This finding suggests that hapsis significantly influences hand preference for grasping and raises the possibility that individuals who have never had a visual experience (but a lifetime of haptic experience) would exhibit little to no hand preference.

A population absent of any visual experience are congenitally blind (CB) individuals. CB refers to an individual who was born without vision (or lost vision shortly thereafter) thus resulting in no recollection of a visual experience [13]. These individuals use their sense of touch hundreds of times per day to identify, manipulate, and pick up objects, and therefore have had extensive experience using haptically-guided grasping. Hand preference for grasping in CB individuals, however, has only been reported anecdotally in children [14]–[17] and these studies have shown mixed results. Hand preference for grasping in an adult CB population remains unknown. Based on the results of Stone and Gonzalez [12] in which a brief haptic experience equalized hand use, one would predict no hand preference for grasping in CB individuals. Or perhaps, even a left-hand preference as several studies have shown a left-hand advantage during the haptic discrimination of objects [1], [18]–[22]. In the present study we investigated these speculations. The *first goal* therefore, was to document hand preference for grasping in an adult CB population.

Hand preference for reaching and grasping is also influenced by the different motor programs that mediate these movements. It has been proposed that planning to reach for an object is guided by its spatial attributes (distance, position), whereas planning to grasp an object is guided by its intrinsic properties (size, shape; [23]). With respect to grasping for example, when picking up the aforementioned apple, the motor program that mediates the grasp would assess its size and shape and transform this information into the appropriate grip type. In this case, most likely a grip that requires all five fingers (i.e. a power grasp) would be used. In contrast, if one were to pick up a grape, its small size would prompt the use of only the thumb and index finger (i.e. a precision grasp). That is, previous research has shown that object size significantly affects hand preference during visually-guided grasping. For example, Vainio et al. [24] found a right-hand preference for precision grips and a left-hand preference for power grips. Similarly, other studies have shown that individuals will use their right hand more often to grasp small as opposed to big objects [4], [25]. It remains to be shown if this pattern persists for grasping without vision. Kinematic studies have shown that, in the absence of vision, visually-unimpaired individuals display disruptions in their movement including larger maximum grip apertures [26]–[29] and slower movement times [30], [31]. Interestingly, this does not seem to be the case for blind individuals. Kinematic analyses have shown that when reaching for an object, blind individuals *do* display differences in movement (i.e. a double opening and closing of the hand towards the target), but they still accurately scale to the size of the unseen object (in a manner similar to sighted individuals) [32], [33]. In light of this evidence, we investigated if object size affects hand *preference* for those with lifelong deprivation of visual experience (CB individuals). Addressing this possibility was the *second goal* of the current study.

CB and visually unimpaired (VU) individuals were tested on the block building task (see [4], [10], [12]). Participants were asked to replicate models from a tabletop of evenly distributed building blocks. Participants built three models using small blocks and three models using big blocks to assess the effect of object size on hand preference. In Experiment One, CB and a group of age- and gender-matched VU participants (VU-Matched controls) were assessed for haptically-guided grasping (all participants were blindfolded). In Experiment Two, visually- and haptically-guided grasping were directly compared in two groups of VU students (VU-Sighted, VU-Blindfolded). In both experiments, hand preference for grasping was documented in ipsilateral (same side as the hand) and contralateral (opposite side of the hand) space as studies have found that reaching across the midline is a powerful indicator of hand preference [6], [8], [34].

## Experiment One

### Methods and Procedures

#### Participants

**Figure 1:**
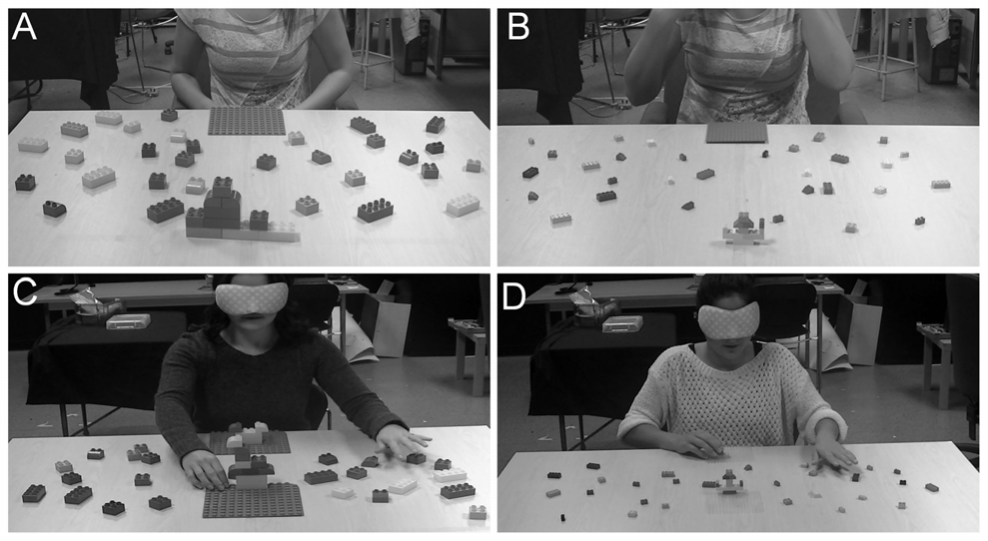
Experimental setup with big blocks (A). Photograph of experimental setup with big blocks scattered across the tabletop. **Experimental setup with small blocks** (**B**)**.** Photograph of experimental setup with small blocks scattered across the tabletop. **Power grasp formation for big blocks** (**C**)**.** Photograph of blindfolded participant exhibiting an extension of all five fingers (resembling a power grasp) while reaching for a big block. **Power grasp formation for small blocks** (**D**)**.** Photograph of blindfolded participant exhibiting an extension of all five fingers (resembling a power grasp) while reaching for a small block.

With respect to the photographs of participants in Figure 1, each individual signed a consent form that read: “The results from this study will be reported in general terms in the form of speech, writing, photograph or video that may be presented in manuscripts submitted for publication in scientific journals, or oral and/or poster presentations at scientific meetings, seminars, and/or conferences.” Participants were therefore aware that their photograph may be used for a published manuscript when their signature was given on the consent form for the current experiment.

A priori power analysis suggested that with power at 0.95, effect size of 0.7, and statistical significance set at 0.05, the current study would require a total sample size of 16 participants (G*Power 3.1.9.2). A total of 24 participants were recruited for this experiment. Furthermore, it should be noted that participant's gender was not balanced, as gender differences have not been reported in earlier studies involving a similar task [25].

Congenitally blind (CB) participants: Ten self-reported right-handed CB individuals (8 males) and two late blind individuals (1 female) were recruited from the Canadian National Institute for the Blind (CNIB) in Lethbridge, Edmonton, Medicine Hat and Calgary (Alberta). These participants were between the ages of 20 and 76 years of age. The two late blind individuals suffered from retitinitis pigmentosa. One had been totally blind for over 50 years and the other for 10 years, although this individual reported suffering from visual deprivation since childhood. Visually-unimpaired (VU)-Matched controls: Twelve self-reported right-handers (9 males) recruited from the Lethbridge area between the ages of 20 and 73 participated. For each CB participant, we tested one VU participant of the same gender and approximately the same age (±3 years). The study was approved by the University of Lethbridge Human Subjects Research Committee (protocol #2011–22) and all participants gave written informed consent in accordance with the Declaration of Helsinki. Participants were naïve to the purposes of the study.

#### Apparatus and Stimuli

*Handedness Questionnaire*: A modified version of the Edinburgh [35] and Waterloo [36] handedness questionnaires were given to all participants at the end of the block building task. This version included questions on hand preference for 22 different tasks. Participants had to rate which hand they prefer to use for each task on a scale +2 (right always) +1 (right usually), 0 (equal), −1 (left usually) and −2 (left always). Each response was scored as 2, 1, −1, or −2 and a total score was obtained by adding all values. Possible scores range from +44 for exclusive right-hand use to −44 for exclusive left-hand use. CB participants were also asked about their diagnosed visual condition, ability to read Braille, and ability to function independently (i.e. yes or no). This information is included in Table 1.

**Table 1:** Information pertaining to each CB and Late Blind participants' (initials included) visual condition, hand used to read Braille, and ability to function independently.

Participant	Visual Condition	Hand used for Braille	Functions independently?
1 BV	Congenital glaucoma	Both	Yes
2 CM	Bilateral Retinoblastoma	Both	Yes
3 WR	Optic nerve hypoplasia	Both	Yes
4 BD	Retinopathy of prematurity	Both	Yes
5 CM	Leber Congenital Amaurosis	Both	Yes
6 EC	Leber Congenital Amaurosis	Both	Yes
7 GE	Optic Atrophy	Both	Yes
8 LP	Retinopathy of prematurity	Right	Yes
9 RG	Optic Nerve Hypoplasia	Unknown	Partial*
10 NS	Retinitis pigmentosa	Unknown	Yes
11 WB	Retinitis pigmentosa	Left	Yes
12 MH	Leber Congenital Amaurosis	Both	Yes

*resides in an assisted living home.

*Block Building Task*: A total of six models built with MEGA BLOKS (big blocks) andLEGO (small blocks) were used for the experiment. Three models were built using big blocks (ranging in size from 3.1 L×3.1 W×2.0 cm H to 6.3 L×3.1 W×2.0 cm H) and three using small blocks (ranging in size from <0.7 L×0.7 W×1.0 cm H to 6.3 L×1.5 W×1.0 cm H). Each model contained 10 blocks of various colours and shapes. Scattered on a table (122 L×122 W×74 cm H with a working space of 70 L×122 W×74 cm H) were all the blocks that made up the three models of each set (i.e. either 30 small blocks or 30 big blocks; see Figure 1A and 1B). The models were prepared ahead of time by the experimenter. The same six models were used with all participants. The same number of blocks were placed on the left and right side of the table. There was a fixed building plate (19L×19 cm W) located within arms' length of the participant. Additionally, there was an exact duplicate of this building plate in the front and center of the participant. The far plate had the model to be replicated attached to it, and the near plate was used for the construction of the new model.

#### Procedures

Participants were seated in front of the table facing the middle of the display that contained either “big blocks” or “small blocks”. Participants were notified that for the next three models they would be building with big *or* small blocks. In other words, participants were never presented with a table that contained small and big blocks at the same time. After the participant was comfortably seated, they were instructed to replicate the model as quickly and accurately as possible from the blocks given on the table. No other instruction was given. All participants were blindfolded prior to and during the task. Therefore, they were only able to use their sense of touch to identify the model and blocks. Once the model was replicated, both models were removed from the table and a new model was given. No blocks were replaced after each model was completed. Six models were built in total. Starting block size (small or big) was counterbalanced among participants. The task was recorded on a JVC HD Everio video recorder approximately 160 cm away from the individual with a clear view of the tabletop, building blocks, and participants' hands.

#### Data Analysis

All recorded videos were analyzed offline. Each grasp was recorded as a left or right hand grasp in the participants' ipsilateral or contralateral space. The total number of grasps was calculated to determine a percent for right-hand use (number of right grasps/total number of grasps X 100). The time in which it took participants to construct each model was recorded on a stopwatch and reported in seconds. Therefore, the dependent variables included percentage of overall right-hand use, percentage of hand use in contralateral space (contralateral grasps), and the time in seconds that it took participants to complete the models. Data were assessed and no violations for homogeneity of variance and normality were found prior to analysis. Partial Eta Squared values were used to show effect size (ES). Data were analyzed using SPSS Statistics 18.0 for Windows (SPSS Inc., Chicago, IL, USA).

### Results (Experiment One)

#### Handedness questionnaire

All participants self-reported as right-handers and this was confirmed by the handedness questionnaire. Congenitally Blind participants: Given that not all 22 questions applied to the CB participants, their scores were normalized to the traditional scale for this questionnaire (−44 to +44) regardless of how many questions they answered. To normalize the scores, we took the participant's total score (e.g. 25) divided by the total number of questions that participant answered (e.g. 19) and this value was then multiplied by 22, which is the total number of possible questions that could have been answered (and the total number of questions all of the VU individuals answered). On average, CB participants received an overall average score of 33.8 (±1.0 SE; range +28 to +41.9) points out of a total possible score of −44/+44. VU-Matched controls: VU-Matched controls participants answered all 22 questions, and received an overall average score of 31.6 (±2.0 SE range +17 to +40) points out of a total possible score of −44/+44. An independent samples t-test revealed that there was no significant difference between the two groups (*t* (22)  = 1.2; *p = *0.2).

#### Hand use for grasping

**Figure 2:**
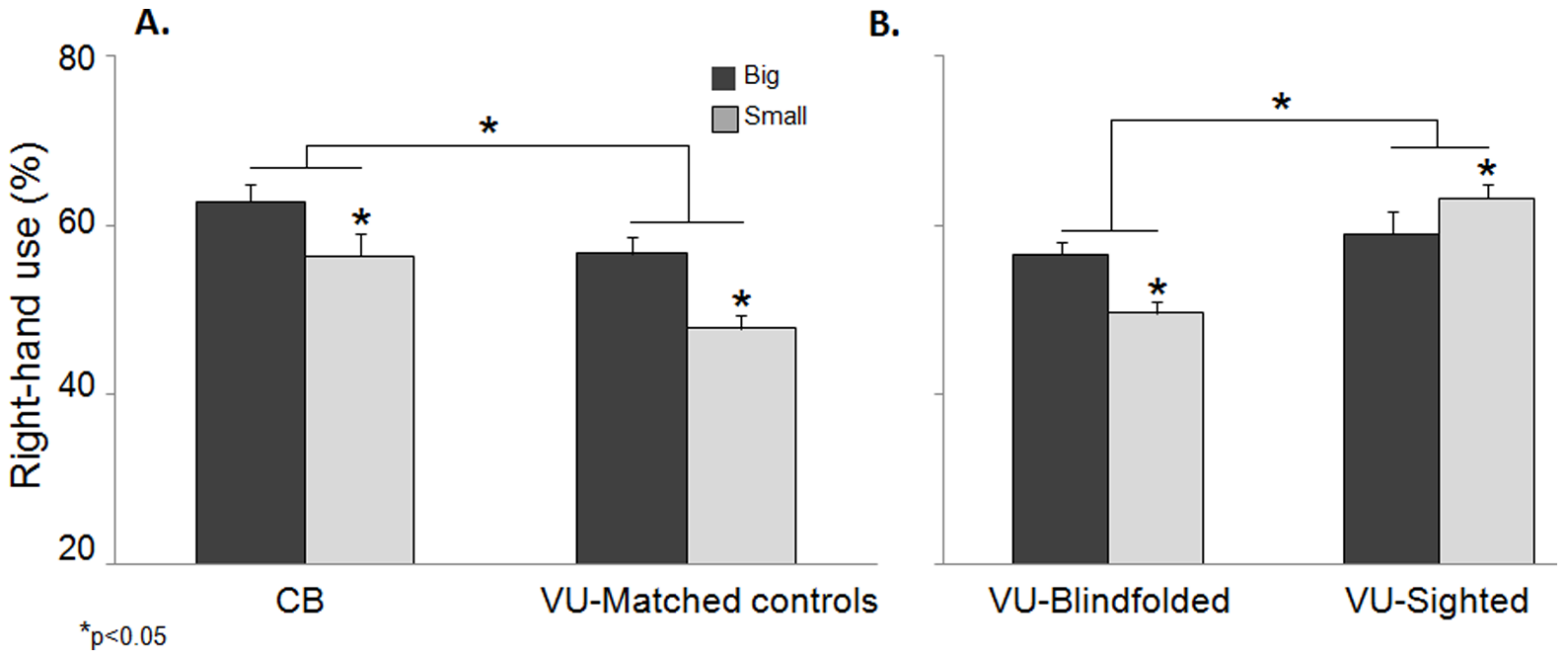
Hand use for grasping for big and small blocks in Experiment One (A). This graph demonstrates right-hand use as a function of object size for the two groups: CB and VU-Matched controls (Experiment One). The dark grey bars represent the big blocks. The light grey bars represent the small blocks. Note the significant difference between the CB and VU-Matched controls, as well as reduction in right-hand use for the small blocks seen in both groups. **Hand use for grasping for big and small blocks in Experiment Two** (**B**)**.** This graph demonstrates right-hand use as a function of object size for the two groups: VU-Blindfolded and VU-Sighted groups (Experiment Two). The dark grey bars represent the big blocks. The light grey bars represent the small blocks. Note the significant difference between the VU-Sighted and VU-Blindfolded participants, as well as the opposite pattern of hand use exhibited for object size within the two groups.

Analysis using a 2 (object size) X 2 (group) repeated measures ANOVA was performed on the percentage of right-hand use for grasping during the task. Object size (big, small) was a within subject factor and group (CB, VU-Matched controls) a between subject factor. Overall, we found a main effect of object size (*F* (1, 22)  = 26.2; *p<*0.0001, ES  = 0.54). Participants used their right hand significantly more to grasp the big blocks (59.6±1.4%) compared to the small blocks (52.0±1.5%). There was also a main effect of group (*F* (1, 22)  = 8.1; *p* = 0.009, ES  = 0.26). Participants in the CB group used their right hands significantly more to grasp the blocks (59.5±1.8%) than the VU-Matched controls (52.2±1.8%), regardless of object size. The interaction between group and size was not significant (*p = *0.42). Both groups displayed the same pattern of hand preference for both big and small blocks. CB participants used their right hand 62.7±2.0% when grasping big blocks and 56.3±2.1% when grasping small blocks. VU-Matched controls used their right hand 56.6±2.0% when grasping big blocks, and 47.8±2.1% when grasping small blocks (see Figure 2A).

*Contralateral grasps*: Analysis using a 2 (object size) X 2 (hand) X 2 (group) repeated measures ANOVA was performed on the percentage of contralateral grasps made during the task. Object size (big, small) and hand (right, left) were within factors and group (CB, VU-Matched controls) the between factor. Analyses did not show a significant effect of object size (*F* (1, 22)  = 0.6; *p = *0.42, ES  = 0.02), therefore data were collapsed across this variable. A 2 (hand) X 2 (group) repeated measures ANOVA was performed on the percentage of contralateral grasps during the task. Hand (right, left) was a within subject factor and group (CB, VU-Matched controls) a between subject factor. There was no main effect of hand (*F* (1, 22)  = 2.8; *p = *0.1, ES  = 0.11). There was no main effect of group (*p* = 0.2). However, the group by hand interaction was significant (*F* (1, 22)  = 5.2; *p = *0.03, ES  = 0.19). Post hoc analysis (paired samples t-test) revealed that the CB group made significantly more right-handed grasps into left contralateral space (6.1±2.0%) than left-handed grasps into right contralateral space (1.6±0.5%; *t* (11)  = 2.2; *p = *0.04). This was not the case for the VU-Matched controls (2.4% versus 3.3%; *t* (11)  =  −0.6; *p* = 0.55).

#### Build times for models

Analysis using a 2 (object size) X 2 (group) repeated measures ANOVA was performed on the average time in seconds it took for participants to complete one model. Object size (big, small) was a within subject factor and group (CB, VU-Matched controls) a between subject factor. Overall, we found a main effect of object size (*F* (1, 22)  = 14.2; *p = *0.001, ES  = 0.39). Participants were faster at completing one model made of big blocks (175.7±11.9s) compared to one model made of small blocks (213.0±14.2). There was no main effect of group (*F* (1, 22)  = 0.8; *p = *0.53, ES  = 0.01). The interaction between size and group reached significance however (*F* (1, 22)  = 4.1; *p = *0.05). Post hoc analysis (paired samples t-test) revealed that VU-Matched controls were significantly faster at completing one model made of big blocks (173.2±12.6s) compared to one model made of small blocks (230.7±21.2s; *t* (11)  =  −3.7; *p = *0.003). No such difference was found in the CB group, who completed one model made of big blocks in approximately the same time as one model made of small blocks (178.1±16.9s and 195.2±20.1s respectively; *t* (11)  =  −1.3; *p* = 0.1).

### Discussion (Experiment One)

Although the majority of the participants were congenitally blind (n = 10), we recognize that two of them were late blind individuals. These individuals, however, reported suffering a lifetime of visual impairment, and had experienced total blindness for a significant number of years. Hand preference for grasping for these individuals was indistinguishable from that of CB individuals, and the results remained unaffected when the two participants are removed, thus they were included in the current study.

Overall the results yielded two interesting findings. First, CB participants preferred their right hand significantly more for grasping the blocks compared to the VU-Matched controls. Second, object size affected hand preference for grasping in both the CB and VU-Matched controls groups. Participants used the right hand more to grasp the big blocks when compared to the small blocks. This effect seen in haptically-guided grasping is opposite to what has previously been reported during visually-guided grasping. Thus, to contrast the effect that object size could have in haptically- versus visually-guided grasping, we tested a group of blindfolded young adult controls (VU-Blindfolded) and a group of sighted young adult controls (VU-Sighted). This last group was presented with the exact same task as the haptically-guided group but they completed the task using vision.

## Experiment Two

### Methods and Procedures

#### Participants

A priori power analysis suggested that with power at 0.95, effect size of 0.7, and statistical significance set at 0.05, the current study would require a total sample size of 16 participants (G*Power 3.1.9.2). A total of 24 participants were recruited for this experiment.

VU-Blindfolded participants: Twelve self-reported right-handers (4 males) from the University of Lethbridge, between the ages of 19 and 31, participated. VU-Sighted participants: Twelve self-reported right-handed individuals (4 males) from the University of Lethbridge, between the ages of 19 and 23, participated. The study was approved by the University of Lethbridge Human Subjects Research Committee (protocol #2011–22) and all participants gave written informed consent in accordance with the Declaration of Helsinki. Participants were naïve to the purposes of the study.

#### Apparatus and Stimuli

All the display material and equipment were the same as Experiment One.

#### Procedures

All procedures were identical to those in Experiment One, with one alteration: those in the VU-Sighted group did not wear a blindfold during the task. In turn, the task was haptically-guided (as in Experiment One) for the VU-Blindfolded group and visually-guided for the VU-Sighted group.

#### Data Analysis

Data analysis was the same as Experiment One. Bonferroni correction was applied to all multiple group comparisons.

### Results (Experiment Two)

#### Handedness questionnaire

All participants self-reported as right-handers and this was confirmed by the handedness questionnaire. VU-Blindfolded: The average score was +30.4 (±1.7 SE; range +21 to +39) out of a total possible score of −44/+44. VU-Sighted: The average score was +32.2 (±1.9 SE; range +22 to +43) out of a total possible score of −44/+44. An independent samples t-test revealed that there was no significant difference between the two groups (*t* (22)  =  −0.7; *p = *0.4).

#### Hand use for grasping

Analysis using a 2 (object size) X 2 (group) repeated measures ANOVA was performed on the percentage of right-hand use for grasping during the task. Object size (big, small) was a within subject factor and group (VU-Sighted, VU-Blindfolded) a between subject factor. There was no main effect of object size (*F* (1, 22)  = 0.4; *p = *0.52, ES  = 0.01) but a main effect of group (*F* (1, 22)  = 13.8; *p* = 0.001, ES  = 0.38). Consistent with Stone and Gonzalez [12], blindfolded participants used their right hand significantly less for grasping (53.0±1.5%) than sighted individuals (61.1±1.5%), regardless of object size. A significant interaction between object size and group was found (*F* (1, 22)  = 17.1; *p*<0.0001, ES  = 0.43). Post hoc analysis (paired samples t-tests) revealed that, just as in Experiment One, VU-Blindfolded individuals used their right hand more to grasp big blocks (56.5±1.5%) compared to small blocks (49.6±1.2%; *t* (11)  = 3.1; *p = *0.009). However, VU-Sighted individuals used their right hand more to grasp small blocks (63.6±1.6%) compared to big blocks (58.6±2.5%; *t* (11)  =  −2.6; *p = *0.02), as shown in Figure 2B. Noteworthy, the values of hand use for the VU-Blindfolded individuals were identical to those of the VU-Matched controls in Experiment One.

*Contralateral grasps*: Analysis using a 2 (object size) X 2 (hand) X 2 (group) repeated measures ANOVA was performed on the percentage of contralateral grasps made during the task. Object size (big, small) and hand (right, left) were within factors and group (VU-Blindfolded, VU-Sighted) the between factor. As in Experiment One, analyses did not show a significant effect of object size therefore data were collapsed across this variable (*F* (1, 22)  = 2.8; *p = *0.1, ES  = 0.11). Therefore, a 2 (hand) X 2 (group) repeated measures ANOVA was performed on the percentage of contralateral grasps during the task. Hand (right, left) was a within subject factor and group (VU-Blindfolded, VU-Sighted) a between subject factor. There was a main effect of hand (*F* (1, 22)  = 28.3; *p<*0.0001, ES  = 0.56). Both groups made significantly more contralateral grasps with the right (6.2±0.9%) versus the left (0.9±0.3%) hand. There was a main effect of group (*F* (1, 22)  = 49.0; *p<*0.0001, ES  = 0.69). The VU-Sighted group made significantly more contralateral grasps than the VU-Blindfolded group (6.0% versus 1.1%, respectively). The group by hand interaction was also significant (*F* (1, 22)  = 21.7; *p<*0.0001, ES  = 0.49). Post hoc analysis (paired samples t-tests) revealed that the VU-Sighted group made significantly more right-handed grasps in the left contralateral space (10.9±1.8%) than left-handed grasps in the right contralateral space (0.8±0.3%; *t* (11)  = 5.2; *p<*0.0001). This was not the case for the VU-Blindfolded group (1.5±.4% versus 0.8±0.3%; *t* (11)  = 1.1; *p* = 0.2).

#### Build times for models

Given that the VU-Sighted group built the models substantially faster than the VU-Blindfolded group (likely due to visual availability; p<0.0001), the two groups were compared separately. A paired samples t-test revealed that VU-Sighted participants completed the task with the big blocks significantly faster than with the small blocks (t (11)  =  −3.8; p = 0.003). Participants completed one model made of big blocks in approximately 22.6±0.9s and one model made of small blocks in 25.7±1.2s. Similarly to the results of Experiment One, the VU-Blindfolded group completed one model made of big blocks significantly faster than one model made of small blocks (t (11)  =  −2.7; p = 0.01; Mean time  = 128.3±9.2s and 153.6±15.4s for big and small blocks respectively).

#### Comparison between Experiment One and Experiment Two

**Figure 3:**
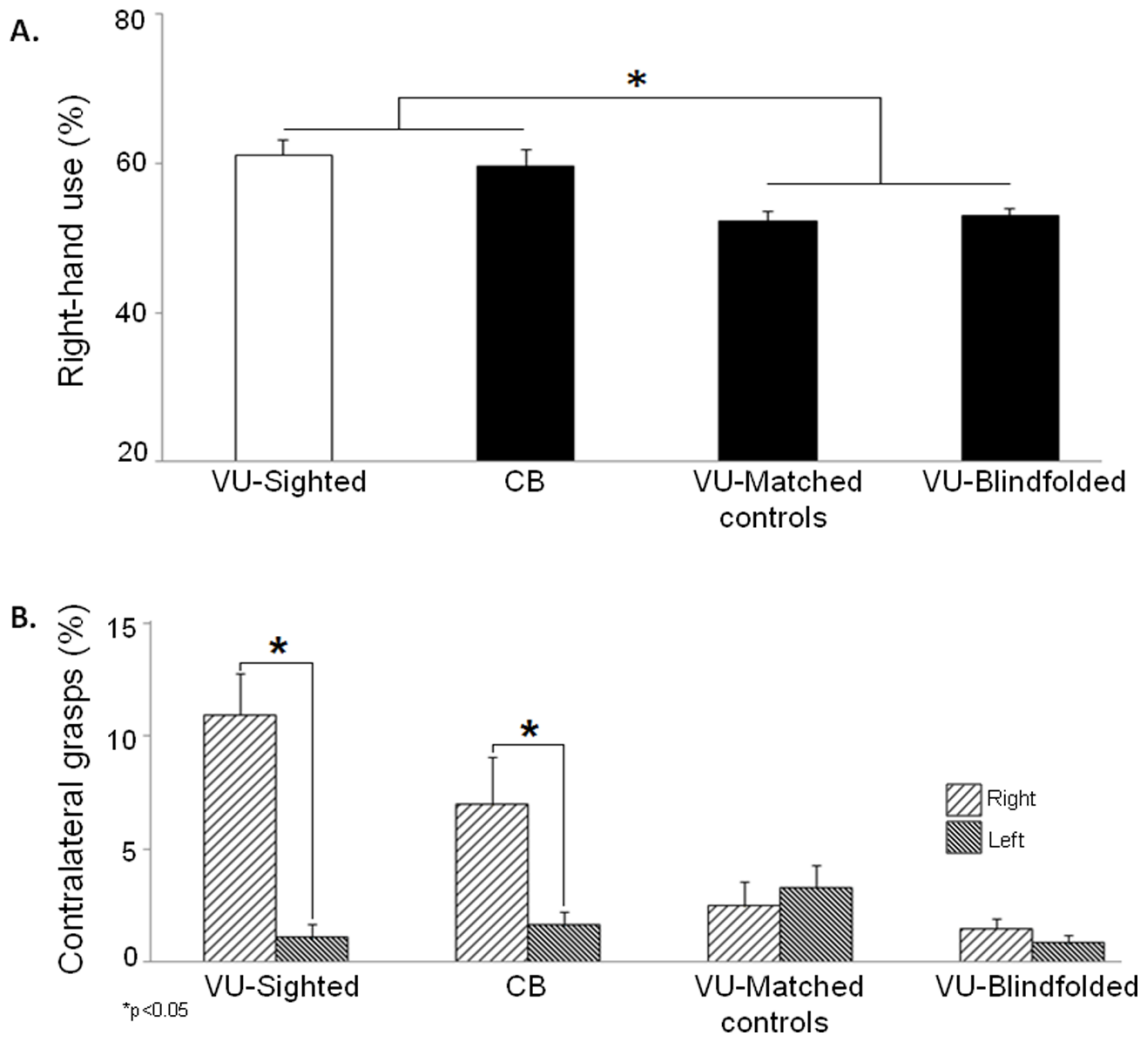
Overall hand use for grasping across all groups (A). This graph demonstrates overall right-hand use (across object size) in percentage for groups in Experiment One (CB, VU-Matched controls) and Experiment Two (VU-Sighted, VU-Blindfolded). White bars signify that the participants had vision for the duration of the task. Black bars signify that the participants had no vision (blind or blindfolded). Note that there is *no* significant different in right-hand use between VU-Sighted and CB participants, but these groups are different from the VU-Blindfolded and VU-Matched controls. **Hand use for contralateral grasps across all groups** (**B**)**.** This graph demonstrates the average percentage of contralateral grasps made with the left and right hand for the VU-Sighted, CB, VU-Matched controls, and VU-Blindfolded groups. The spaced rightward diagonal bars represent the right hand and the compressed leftward diagonal bars represent the left hand. Note the significant difference in contralateral grasps made with each hand for the VU-Sighted and CB groups but not for the VU-Matched controls. Note also, that the VU-Sighted and CB groups behaved similarly.

We performed an analysis using a 2 (object size) X 4 (group) repeated measures ANOVA on the percentage of overall right-hand use for grasping. Object size (big, small) was a within subject factor and group (CB, VU-Matched controls, VU-Blindfolded, and VU-Sighted) a between subject factor. We found a main effect of size (*F* (1, 44)  = 17.0; *p<*0.0001, ES  = 0.28). Again, participants used the right hand more often to grasp big (58.6±1.0%), as opposed to small (54.3±0.9%) blocks. There was also a main effect of group (*F* (3, 44)  = 7.1; *p<*0.0001, ES  = 0.32). The CB group used their right hands significantly more for grasping than the VU-Blindfolded (*p* = 0.05) and the VU-Matched controls (*p* = 0.02). Interestingly, however, the CB group behaved similarly to the VU-Sighted group (*p = *1.0). That is, both groups used their right hand to the same extent during the grasping task. Importantly, as depicted in Figure 3A, the VU-Matched controls and VU-Blindfolded groups differed from the VU-Sighted group (*p* = 0.003; *p* = 0.009, respectively). The VU-Matched controls and VU-Blindfolded group did not differ from each other (*p* = 1.0). The size by group interaction was also significant (*F* (3, 44)  = 9.2; *p<*0.0001, ES  = 0.38). Post hoc analysis (independent samples t-test) revealed that the VU-Sighted group used the right hand significantly more than the VU-Blindfolded (*t* (22)  =  −6.7; *p<*0.0001), VU-Matched controls (*t* (22)  =  −6.9; *p<*0.0001), and CB (*t* (22)  =  −2.3; *p* = 0.03) groups when grasping small blocks. This was not the case for the big blocks, however (all comparisons *p*>0.1). This result reflects the different effect that object size poses on haptically- versus visually-guided grasping, particularly for small objects. In other words, small objects were more often picked up with the right hand during visually-guided, but less often during haptically-guided grasping. This is not surprising as we have previously shown less pronounced right-hand use for grasping big blocks [4], [25].

*Contralateral grasps*: Analysis using a 2 (object size) X 2 (hand) X 4 (group) repeated measures ANOVA was performed on the percentage of contralateral grasps made during the task. Object size (big, small) and hand (right, left) were within factors and group (CB, VU-Matched controls, VU-Blindfolded, VU-Sighted) the between factor. Again, analyses did not show a significant effect of object size therefore data were collapsed across this variable (*F* (1,44)  = 3.0; *p = *0.1, ES  = 0.05). Therefore, a 2 (hand) X 4 (group) repeated measures ANOVA was performed on the percentage of contralateral grasps during the task. Hand (right, left) was a within subject factor and group (CB, VU-Matched controls, VU-Blindfolded, VU-Sighted) a between subject factor. There was a main effect of hand (*F* (1, 44)  = 20.1; *p<*0.0001, ES  = 0.31). Participants made significantly more right-handed grasps in the left contralateral space (5.4±0.7%) compared to left-handed grasps in the right contralateral space (1.7±0.3%). In other words, participants were more likely to reach across to the left contralateral space to grasp a block with the right hand compared to reaching into the right contralateral space to grasp a block with the left hand. There was a main effect of group (*F* (3, 44)  = 6.4; *p = *0.001, ES  = 0.30). The VU-Sighted group made significantly more right-handed grasps in left contralateral space than the VU-Blindfolded (*p* = 0.001) and the VU-Matched controls (*p* = 0.05). No differences emerged between the VU-Sighted and CB groups (*p* = 0.8). The hand by group interaction was also significant (*F* (3, 44)  = 8.4; *p<*0.0001, ES  = 0.36). Post hoc analysis (paired samples t-tests) revealed that the VU-Sighted group made significantly more right-handed grasps in the left contralateral space (10.9±1.8%) than left-handed grasps in the right contralateral space (1.0±0.5%; *t* (11)  = 5.2; *p<*0.0001). The CB also made significantly more right-handed grasps in the left contralateral space (6.1±2.0%) than left-handed grasps in the right contralateral space (1.6±0.5%; *t* (11)  = 2.2; *p = *0.04). However, this was not the case for the VU-Matched controls (2.4±1.0% versus 3.3±0.9%; *t* (11)  =  −0.6; *p = *0.55) nor for the VU-Blindfolded group (1.5±1.5 versus 0.8±1.2%; *t* (11)  = 1.1; *p = *0.27; see Figure 3B). Furthermore, independent samples t-test revealed that the VU-Matched controls made significantly more left-handed grasps in the right contralateral space than the VU-Blindfolded (3.3% versus 0.8%; *t*(22)  = 2.4; *p* = 0.02). All remaining comparisons between hand and group were not significant. To emphasize, independent samples t-test results showed no significant difference in percentage of right-handed grasps completed in the left contralateral space between the CB and the VU-Sighted group (*t* (22)  =  −1.4) *p*>0.1). Thus, in terms of grasping in contralateral space, the CB group displayed similar behaviour to the VU-Sighted group.

## General Discussion

The present study had two main goals: first, to document hand preference for grasping in a congenitally blind (CB) population and second, to investigate how object properties, specifically object size, would influence hand preference during haptically-guided grasping. CB and visually unimpaired (VU: Sighted and Blindfolded/Matched controls) individuals were asked to replicate 3D models using small or large blocks scattered on a tabletop. Results showed that CB individuals exhibit a right-hand preference for grasping, and this preference was significantly greater than the VU-Matched controls and VU-Blindfolded group. Furthermore, this right-hand preference in CB individuals was not different from the VU-Sighted group, which was further confirmed by the analysis of contralateral grasps. These results suggest that, regardless of visual experience, hand preference for gasping is lateralized to the right hand. A second finding was the influence that object size exerted on hand preference. Consistent with previous studies [4], [25] VU-Sighted participants used their right hand more often to grasp the small, as opposed to the big blocks. However, when blindfolded, VU participants demonstrated the opposite pattern: they preferred their right hand to grasp the big, versus the small, blocks. This result was also seen in the CB participants. Overall, these findings suggest first, a left-hemisphere specialization for grasping regardless of lifetime visual *or* haptic experience; and second, that object properties such as size pose different demands on visually- versus haptically-guided grasping.

Studies exploring hand use in blind individuals have focused on haptically-guided tasks such as reading Braille [37]–[39] or identifying: geometrical shapes [2], [40], tactile pictures [41]–[43], or spatial targets [44]. These studies have highlighted the ability, and in some cases, the advantage that CB individuals exhibit over VU participants during tactile recognition tasks. In some studies, this advantage was specific to one hand. For example, when CB participants were asked to read Braille they were more proficient when using their left hand [37]. Another study showed that when sorting different objects, CB individuals were faster with their left hand than VU participants were with either hand [16]. None of these studies however, specifically investigated hand preference for grasping in CB adults. In the present investigation, we document CB individuals' right-hand preference for grasping. Interestingly, this right-hand preference was similar to that of sighted individuals when using vision. A previous study has shown a reduction of right-hand use during haptically-guided grasping in VU individuals [12] which was replicated in the current study. CB participants however, used their right hand to the same extent as the sighted VU participants. In fact, the analysis of contralateral grasps confirmed that CB behave virtually identical to VU-Sighted individuals in terms of hand use. When reaching across the midline, both groups showed a marked right-hand preference over the left for grasping the blocks. These results suggest that the mechanisms of hand preference for grasping develop *independently* of visual experience. In an investigation of hand preference and ability in CB children, Ittyerah suggests that hand preference might not be determined by vision [17]. Furthermore, based on 39 neuroimaging studies, Ricciardi et al. [45] argue that brain functional organization is to a large extent independent from visual experience. For example, sighted and CB individuals were asked to tactically recognize different objects (bottles, shoes, and masks of faces) while brain activation was measured through functional MRI [46]. The results showed that category-relative object activation in occipito-temporal areas (ventral stream), was similar for sighted and CB individuals. Likewise, overlapping activation patterns in the dorsal stream have been shown during kinaesthetically guided hand movements in both sighted and CB individuals [47]. Our results of hand use during grasping are in line with these previous studies in that CB individuals behaved virtually identical to sighted individuals.

Another finding of the present investigation was the effect that object size exerted on hand preference during grasping. Studies have shown that the kinematics of prehensile movements are contingent upon object size. Movement time, peak velocity and grip apertures all respond to changes in object size. For instance, the larger the object to be grasped, the longer it takes to reach maximum grip aperture [26], [48], [49]. When reaching for smaller objects, individuals exhibit lower and earlier peak velocities and longer deceleration phases when compared to reaching for larger objects [50]–[53]. Interestingly, grip scaling in blind individuals is accurate and indistinguishable from sighted individuals when reaching for different sized objects [32]. These results illustrate how object size influences kinematics of reaching and grasping, even in the absence of vision. With respect to hand preference, previous investigations [4], [25] have also highlighted the influence of object size and have shown that when grasping small objects, there is an increase in right-hand use. In the current investigation we replicated this finding for visually-guided grasping. Interestingly, during haptically-guided grasping, both CB and VU individuals used the right hand more often for grasping the large objects. This finding suggests that object characteristics affect patterns of hand use differently for haptically- versus visually-guided grasping.

We offer two non-mutually exclusive explanations for this phenomenon. First, it is possible that, as we have argued before [12], hand use during haptically-guided grasping could be influenced by the known advantage of this hand for haptic discrimination. Several studies have shown a left-hand advantage for object discrimination when guided by hapsis [1], [18], [54]–[61]. For instance, Benton et al. [54] applied tactile stimulation to the hands of individuals and asked them to identify the direction of the stimulus. Participants were significantly more accurate when the stimulation was applied to their left hand. In a series of experiments, Fagot and colleagues have demonstrated a left-hand superiority for recognizing shapes when given a discrimination task. Accordingly, they also found a left-hand advantage in terms of overall time the left hand explored the given objects for these tasks [56], [62], [63]. Fagot's results demonstrate that the left hand is better at encoding haptic information. Support for this evidence was provided via personal communication with the CB individuals in the present study. Upon reviewing the CB testimonials collected during and after our visit we encountered two statements that speak to this matter. When asking the participants if one hand was better for touch, one individual responded, “Well… I might look for more detail with the left”. A second CB individual responded to the question similarly: “My left hand is more focused towards the two fingers, feeling the spatial [properties of the object], and I use my right hand to pick up [the object].” It appears then, that there is a division of labour between the hands when identifying (left hand) and grasping (right hand) an object that one cannot see.

The second possibility that could have contributed to the object size effect is the initial grasp formation. A previous study has shown facilitation of right-handed responses for pincer grasps (i.e. thumb and index finger) and facilitation of left-handed responses for power grasps [24]. Similarly, it has been shown that visually-guided grasps requiring more precision (pincer grasping) are more often executed with the right hand as opposed to grasps that require the whole hand (i.e. power grasps), even in left-handers [4], [25], [64]. In the current experiments we observed that all haptically-guided reaching movements (regardless of the block's size) exhibited an extension of all five fingers (see Figure 1C and 1D) resembling a power grasp (see [65] for similar results). It is possible that this power grasp formation, which favours left-handed grasps, affected the selection of hand use while grasping the small blocks. In fact, Overvliet et al. [66] found that individuals were better at localizing tactile stimuli when the fingers were spread apart (similar to a power grasp formation) compared to when they were close together. We speculate that the decrease in right-hand use seen for haptically-guided grasping stems in part from an increase of left-hand use during haptic discrimination, particularly for the small objects. Although this might not explain the increase in right-hand use seen when grasping the *big* blocks, it provides a preliminary explanation for change in hand use for grasping the small blocks. Furthermore, it remains unknown if these findings would persist in a population of left-handed individuals, which is a limitation of the current study. These possibilities warrant further investigation.

In conclusion, the results indicate that visual availability, but not visual experience, determines hand use in response to object size. Furthermore, the results suggest a left-hemisphere/right-hand specialization for grasping, regardless of visual experience.
